# Antitumor effects of natural killer cells derived from gene-engineered human-induced pluripotent stem cells on hepatocellular carcinoma

**DOI:** 10.1007/s00262-025-03940-5

**Published:** 2025-02-04

**Authors:** Mayuna Nakamura, Yuka Tanaka, Keishi Hakoda, Masahiro Ohira, Tsuyoshi Kobayashi, Kenji Kurachi, Kouichi Tamura, Hideki Ohdan

**Affiliations:** 1 Department of Gastroenterological and Transplant Surgery, Graduate School of Biomedical and Health Sciences, Hiroshima University, 1-2-3 Kasumi, Minami-Ku, Hiroshima, 734-8551 Japan; 2 Division of Regeneration and Medicine, Medical Center for Translational and Clinical Research, Hiroshima University Hospital, 1-2-3 Kasumi, Minami-Ku, Hiroshima, 734-8551 Japan; 3 Kobe Research Institute, HEALIOS K.K., Kobe, Japan

**Keywords:** Hepatocellular carcinoma, iPS cells, NK cells, Antitumor effect, Genetic engineering, Cell therapy

## Abstract

Mortality and recurrence rates of hepatocellular carcinoma (HCC) remain high despite the use of various treatment methods. Recently, cell-based immunotherapy using natural killer (NK) cells has attracted considerable attention in cancer immunotherapy. NK cells generated from induced pluripotent stem cells (iPSCs) are a new option for use as an NK cell resource. The eNK cells (HLCN061, developed by HEALIOS K.K.) are human iPSC-derived NK cells differentiated from clinical-grade iPSCs in which IL-15, CCR2B, CCL19, CD16a, and NKG2D have been introduced. In this study, we aimed to evaluate the potential of eNK cell therapy for HCC treatment. The analysis of eNK cells for cell surface and intracellular molecules revealed that antitumor-related surface molecules (TRAIL, CD226, and CD16) and intracellular cytotoxic factors (perforin, granzyme B, TNFα, and IFNγ) were highly expressed. In addition, eNK cells exhibited high cytotoxicity against HCC cell lines (HepG2, HuH7, and SNU-423), which are sensitive to NKG2D, TRAIL, and CD226. The TRAIL and perforin/granzyme B pathways are largely involved in this cytotoxic mechanism, as indicated by the reduction in cytotoxicity induced by TRAIL inhibitory antibodies and concanamycin A, which inhibits perforin/granzyme B-mediated cytotoxicity. Our data suggest that eNK cells, whose functions have been enhanced by genetic engineering, have the potential to improve HCC treatment.

**Supplementary Information:**

The online version contains supplementary material available at 10.1007/s00262-025-03940-5.

## Introduction

Cell-based immunotherapy has received considerable attention, and various cancer therapeutic approaches have been developed over the past several decades. Natural killer (NK) cells are innate immune cells that account for approximately 15% of circulating blood lymphocytes [1]. NK cells possess various functional factors and target abnormal cells, such as cancer and virus-infected cells, without prior sensitization. This feature has attracted attention because of its applications in cancer immunotherapy. NK cell products can be generated from multiple sources, such as peripheral and umbilical cord blood, NK cell lines, and induced pluripotent stem cells (iPSCs) [2]. Compared with blood-derived NK cells and NK cells derived from iPSCs can be cultured on a large scale and manufactured off-the-shelf. In addition, because these cells are more easily transduced than blood-derived NK cells are, the development of NK cells with enhanced functions, such as transgenic NK and chimeric antigen receptor (CAR)-NK cells, is currently underway.

The iPSCs represent a new option for NK cell generation, and several generation methods have been described [3, 4]. To enhance their functions, iPSC-derived NK cells expressing functional molecules and CARs have been developed using genetically engineered technologies [5–7]. In preclinical studies, iPSC-NK cells have shown effector cytotoxic responses in vitro against a variety of hematological and solid tumor cell lines, including lung, hepatocellular, and ovarian cancers, as well as myeloid leukemia and melanoma [3, 8]. Clinical trials using iPSC-derived NK and CAR-NK cells, alone or in combination with other drugs, have been conducted, and some data have provided promising results [6–8].

Liver cancer is the sixth most common malignancy and third most common cause of cancer-related deaths worldwide [9]. Moreover, hepatocellular carcinoma (HCC) recurrence occurs in approximately 40–80% of patients within five years of hepatic resection or radiofrequency ablation [10]. Although there are various treatment options for HCC, such as surgical resection, liver transplantation, thermal ablation, transarterial chemoembolization, and drugs, new therapeutic options are needed to improve treatment effects and reduce recurrence. Recent studies have shown that targeting NK cells can help in the treatment of HCC, the restoration of normal liver function, and to subsequently increase survival rates of patients with HCC [11]. We previously reported that activated donor liver-derived NK cells are effective in preventing the postoperative recurrence of liver cancer, and the main mechanisms involved were the high expression of tumor necrosis factor (TNF)-related apoptosis-inducing ligand (TRAIL), perforin activity, and interferon (IFN)γ production [12–14]. However, because liver-derived NK cells can only be obtained from liver transplant donors, they are difficult to apply to liver cancer treatment. Other research groups have also reported that peripheral blood NK cells activated by various cytokines [15], glypican 3 (GPC3)-specific CAR-engineered NK cells [16], NK cells expressing the NKG2D-CD3ζ-DAP10 receptor [17], and NK cells with TLR7/TLR8 agonists [18] are effective for HCC treatment [11, 19]. Clinical trials using NK cells alone or in combination with other drugs have been conducted for HCC but have not yet reached practical applications [20]. Therefore, the development of NK cells with high therapeutic efficacy for HCC is required.

In this study, we aimed to evaluate the potential of eNK cells (HLCN061; developed by HEALIOS K.K.), which are human iPSC-derived NK cells differentiated from clinical-grade iPSCs in which IL-15, CCR2B, CCL19, CD16a, and NKG2D have been introduced, in the treatment of HCC.

## Materials and methods

### Generation of eNK cells

The iPSC line was derived from human umbilical cord blood CD34^+^ cells according to published protocols [21] established in the Lonza Walkersville facility (Lonza Laboratories, Walkersville, MD, USA). The eNK cells overexpressing CCL19, CCR2B, FCGR3A (CD16), IL-15, KLRK1 (NKG2D), and HSCT (DAP10) were generated by HEALIOS K.K. [22]. For further details, the genes encoding CCL19, CCR2B, FCGR3A, IL-15, KLRK1, and HSCT were ectopically expressed under the control of the human EF1a promoter using the piggyBac system. Single iPSC clones were obtained through serial dilution, expanded, and cryopreserved. The expression of the transgenes was confirmed via flow cytometry and enzyme-linked immunosorbent assay (ELISA). Gene-modified clones expressing all transgenes were selected and underwent NK cell differentiation.

To introduce, six genes into human-induced pluripotent stem cells (hiPSCs), equimolar amounts of six donor-plasmid transgene expression vectors (totaling 5 μg) and 5 μg of hPBase expression vectors were electroporated into 1.0 × 10^6^ hiPSCs. This was done using the Neon transfection system (Thermo Fisher Scientific) with Preset Program No. 12 (1200 V, 40 ms, 1 pulse). The transfected hiPSCs were then cultured in a complete AK03N medium (Ajinomoto) supplemented with puromycin, hygromycin, and zeocin (Thermo Fisher Scientific) for one week to select cells expressing all transgenes. After selection, the cells were dissociated into single cells and seeded into 96-well plates at a density of one cell per well. They were cultured for approximately two weeks. Candidate clones were then expanded and cryopreserved for future use. Protein expression of the transgenes in the gene-modified hiPSC clones was confirmed using flow cytometry to detect the expression of CCR2B, CD16, and the NKG2D-DAP10 complex. Additionally, enzyme-linked immunosorbent assays (ELISA) were performed to measure the expression of CCL19 and IL-15. For the detection of CCR2B, CD16, and NKG2D-DAP10, the cells were stained with the following antibodies: PE Mouse Anti-Human CD16 (clone 3G8: BD Biosciences, CA, USA), APC-anti-human CD314 (clone 1D11, BioLegend, CA, USA), and APC/Fire 750 anti-human CD192 (clone K036C2, BioLegend). To measure the expression of CCL19 and IL-15, the culture supernatants were analyzed using a MIP3b Human ELISA kit (Abcam, Cambridge, UK) and a Human IL-15 Quantikine ELISA kit (R&D Systems). Karyotype analysis was performed by Sumika Chemical Analysis Service, Ltd., based in Tokyo, Japan. Gene-modified clones expressing all transgenes were selected and underwent NK cell differentiation.

The dissociated gene-modified hiPSCs were cultured in AK03N medium supplemented with 10 μM Y-27632 (FUJIFILM Wako Pure Chemical, Osaka, Japan) for 5 days to form spheroids. The spheroids were then collected and cultured in DMEM/F12 medium (Thermo Fisher Scientific) supplemented with 2 μM CHIR99021 (FUJIFILM Wako Pure Chemical), 80 ng/mL BMP4 (FUJIFILM Wako Pure Chemical or PeproTech), 80 ng/mL VEGF165 (FUJIFILM Wako Pure Chemical), 50 ng/mL bFGF (FUJIFILM Wako Pure Chemical), and 10 μM Y-27632 (FUJIFILM Wako Pure Chemical) for 2 days. This was followed by culturing in Essential 6 medium (Thermo Fisher Scientific) supplemented with 80 ng/mL VEGF165 (FUJIFILM Wako Pure Chemical), 2 μM SB431542 (FUJIFILM Wako Pure Chemical), 50 ng/mL SCF (FUJIFILM Wako Pure Chemical or PeproTech), 50 ng/mL bFGF (FUJIFILM Wako Pure Chemical), and 10 μM Y-27632 (FUJIFILM Wako Pure Chemical) for 2 more days to induce differentiation into a definitive hematopoietic lineage.

Next, the spheroids were cultured in HPCM0002 medium (Kohjin Bio, Saitama, Japan) supplemented with 1 × GlutaMax (Thermo Fisher Scientific), 50 ng/mL SCF (FUJIFILM Wako Pure Chemical or PeproTech), 50 ng/mL IL-3 (PeproTech), and 50 ng/mL FLT3L (PeproTech) for 10 days. Once floating single cells appeared, the spheroids were transferred into AIM-V medium (Thermo Fisher Scientific) supplemented with 5% FBS (SAFC Biosciences, KS, USA), 50 ng/mL SCF (FUJIFILM Wako Pure Chemical or PeproTech), 50 ng/mL FLT3L (PeproTech), 50 ng/mL IL-7 (PeproTech), and 50 ng/mL IL-15 (PeproTech). During NK cell differentiation, the expression of CD56 was monitored. Once the proportion of CD56-positive cells reached 80% of the total population, the eNK cells were cryopreserved. Cryopreserved eNK cells were thawed in a 37 °C water bath and subsequently cultured in CTS AIM-V medium supplemented with 5% FBS, 50 ng/mL SCF, and 50 ng/mL IL-15 for three days prior to use.

### Cell lines and cell culture

HepG2, C3A, SK-HEP-1, PLC/PRF/5, SNU-387, SNU-423, SNU-449, and NK-92 cells were purchased from the American Type Culture Collection (ATCC, Manassas, VA, USA), and HuH7 cells from the Japanese Cancer Research Resources Bank (JCRB, Osaka, Japan). HepG2, C3A, SK-HEP-1, and PLC/PRF/5 cells were cultured in high-glucose Dulbecco’s minimum essential medium (Gibco, Grand Island, NY, USA) supplemented with 10% fetal bovine serum (FBS; Biological Industries, Israel), 1% HEPES buffer (Gibco), and 1% penicillin–streptomycin (PS; Gibco). SNU-387, SNU-423, and SNU-449 cells were cultured in RPMI-1640 medium (Nacalai Tesque, Kyoto, Japan) supplemented with 10% FBS, 1% HEPES buffer, and 1% PS. HuH7 cells were cultured in low-glucose Dulbecco’s minimum essential medium (Sigma-Aldrich, St. Louis, MO, USA) supplemented with 10% FBS and 1% PS. NK-92 cells were cultured in α-minimum essential medium (Nacalai Tesque) composed of 20% FBS, 1% PS, 0.05 mM 2-mercaptoethanol (Nacalai Tesque), and 10 ng/mL interleukin (IL)-2 (Miltenyi Biotec, Bergisch Gladbach, Germany).

### Isolation of human NK cells

Peripheral blood mononuclear cells (PBMCs) were separated from healthy volunteers using Lympholyte®-H cell separation media (Cedarlane Laboratories Ltd., Ontario, Canada). PBNK cells were isolated using an NK cell isolation kit (Miltenyi Biotec), according to the manufacturer’s protocol. NK cell purity was confirmed by flow cytometry that CD56^+^CD3^−^ cells were > 90%. This study was approved by the Ethics Committee of Hiroshima University (No. E2012-9977).

### Flow cytometry

All analyses were performed using a FACS Celesta or FACS Canto II cytometer (BD Biosciences, San Jose, CA, USA). The eNK cells, NK-92 cells, and liver cancer cell lines were stained with the following monoclonal antibodies (mAb). FITC-anti-CD56 (clone B159), APC-H7-anti-CD3 (clone SK7), APC-anti-programmed death 1 (PD-1) (clone MIH4), anti-NKG2D (clone 1D11), anti-TRAIL (clone RIK-2), Alexa Fluor 647-anti-CD226 (clone DX11), BV480-anti-CD16 (clone 3G8), BV421- anti-NKp30 (clone p30-15), BV605-anti-NKp44 (clone p44-8), PE-anti-NKp46 (clone 9E2/Nkp46), anti-CD261 (clone S35-934), anti-CD47 (clone B6H12), anti-PD-L1 (clone MIH1), and anti-PD-L2 (clone MIH18) mAb were obtained from BD Biosciences. APC-anti-MICA/B (clone 6D4), anti-CD112 (clone TX31), PE-anti-CD155 (clone SKll.4), Alexa Fluor 700-anti-NKG2A (clone S19004C), anti-signal regulatory protein-α (SIRPα) (clone 15–414) mAb were obtained from BioLegend (San Diego, CA, USA). PE-anti-CD262 (clone DJR2-4) and anti-CD263 (clone DJR3) mAb were purchased from eBioscience (Carlsbad, CA, USA). APC-anti-ULBP1 (clone #170,818), anti-GPC3 (clone #307,801), PE-anti-ULBP2/5/6 (clone #165,903), anti-ULBP3 (clone #166,510), anti-ULBP4 (clone #709,116), and anti-CD264 (clone #104,918) mAb were purchased from R&D Systems (Minneapolis, MN, USA). Dead cells were excluded from the analysis using forward scatter and 7-amino-actinomycin D (7-AAD; BD Biosciences). Data analyses were performed using FlowJo software ver.10 (BD Biosciences).

### Intracellular flow cytometry

Perforin, granzyme B, IFNα, IFNγ, and TNFα production in NK-92 and eNK cells were measured through intracellular staining, according to the manufacturer's instructions. Briefly, 4 h after treatment with a leukocyte activation cocktail (BD GolgiPlug; BD Biosciences), the eNK and NK-92 cells were stained with FITC-anti-CD56 (clone B159) and APC-anti-CD3 (clone HIT3a) (BD Biosciences) mAb. The cells were then fixed, permeabilized with Cytofix/Cytoperm solution (BD Biosciences), and washed with Perm/Wash Buffer (BD Biosciences). Subsequently, the cells were stained with PE-anti-perforin (clone dG9) (BioLegend), anti-granzyme B (clone GB11), anti-IFNα (clone 7N4-1), anti-IFNγ (clone B27), and anti-TNFα (clone 6401.1111) (BD Biosciences) mAb, and were analyzed using a FACS Canto II cytometer. Data analyses were performed using FlowJo software ver.10.

### CD107a degranulation assay

CD107a degranulation assays were performed as previously described [23]. Briefly, 1 × 10^5^ cells of eNK cells were seeded into round-bottomed 96-well and co-cultured with HepG2, HuH7, or SNU-423 cells at a 1:1 ratio with or without Anti-CD107a (clone H4A3, BD Biosciences) mAb at 37 °C and 5% CO_2_. The control group was cultured with only eNK cells, without hepatocellular carcinoma cells. After one hour of incubation, GolgiStop (BD Biosciences) and GolgiPlug (BD Biosciences) were added to all samples and incubated for a further three hr. Non-adherent cells were collected, washed, stained with anti-CD56 mAb, and analyzed using FACS Canto II cytometer (BD Biosciences). For the confirmation of CD107a expression, dead cells were excluded from analysis using forward scatter and 7-AAD. The expression of IFNγ and TNFα was analyzed by staining with anti-IFNγ (clone B27) and anti-TNF-α (clone 6401.1111) (BD Biosciences) mAB after fixation and permeabilization with Cytofix/Cytoperm solution (BD Biosciences) and washing with Perm/Wash Buffer (BD Biosciences). Data analyses were performed using FlowJo software ver.10.

### Cytotoxicity assay

Cytotoxicity assays were conducted using a real-time cell analyzer (xCELLigence S16 system; Agilent Technologies, Santa Clara, CA, USA). All experiments were performed at 37 °C with 5% CO_2_. Initially, 50 µL culture medium was added to a 16-well E-plate (Agilent Technologies) to measure the background. Then, HepG2, HuH7, or SNU423 cells were seeded onto an E-plate at 4 × 10^4^, 6 × 10^3^, or 5 × 10^3^ cells suspended in 100 µL culture medium, respectively. To ensure uniform cell adhesion, the plates were incubated for 30 min at room temperature and cultured overnight. After incubation, 100 µL of the medium was removed, and NK-92 or eNK cells added at effector/target (E/T) ratios = 1, 5, and 10 (duplicates for each), and for PBNK cells were added at effector/target (E/T) ratios = 1 and 5 (single for each). The plate was allowed to stand for 30 min at room temperature and then set in the device. The cell index was measured every 15 min for 12 h. % Cytotoxicity was calculated using the following formula: % Cytotoxicity = [1 − (cell index of effector and target cells − cell index of effector only)/cell index of target only] × 100.

### Blocking assay

Blocking assays were performed using the ^51^Cr-release assay method, as previously described[24]. Briefly, 1 × 10^4^ or 1 × 10^5^ eNK cells were preincubated at 37℃ in round-bottomed 96-well microtiter plates in the presence of 50 ng/mL IL-15, 10 μg/mL anti-TRAIL mAb, and/or 25 nmol/L concanamycin A (CMA) (Sigma-Aldrich) for 30 min. Then, 1 × 10^4 51^Cr-labeled target tumor cells (HepG2, HuH7, and SNU-423) were added and co-cultured for 4 and 18 h. The percentage of specific ^51^Cr release was calculated as follows:

% Cytotoxicity = [(cpm of experimental release – cpm of spontaneous release)/(cpm of maximum release – cpm of spontaneous release)] × 100.

All assays were performed in quadruplicate.

### Statistical analysis

Data were analyzed using a one-tailed Student’s t-test. Significance differences are indicated as *p < 0.05, **p < 0.01, and ***p < 0.001.

## Results

### eNK cells highly express antitumor molecules

Flow cytometry analysis of the eNK cells after recovery culture from cryopreservation revealed that the CD56^+^CD3^−^ and CD56^dim^CD3^−^ NK cell fractions were approximately 80% and 20%, respectively.

**Fig. 1:**
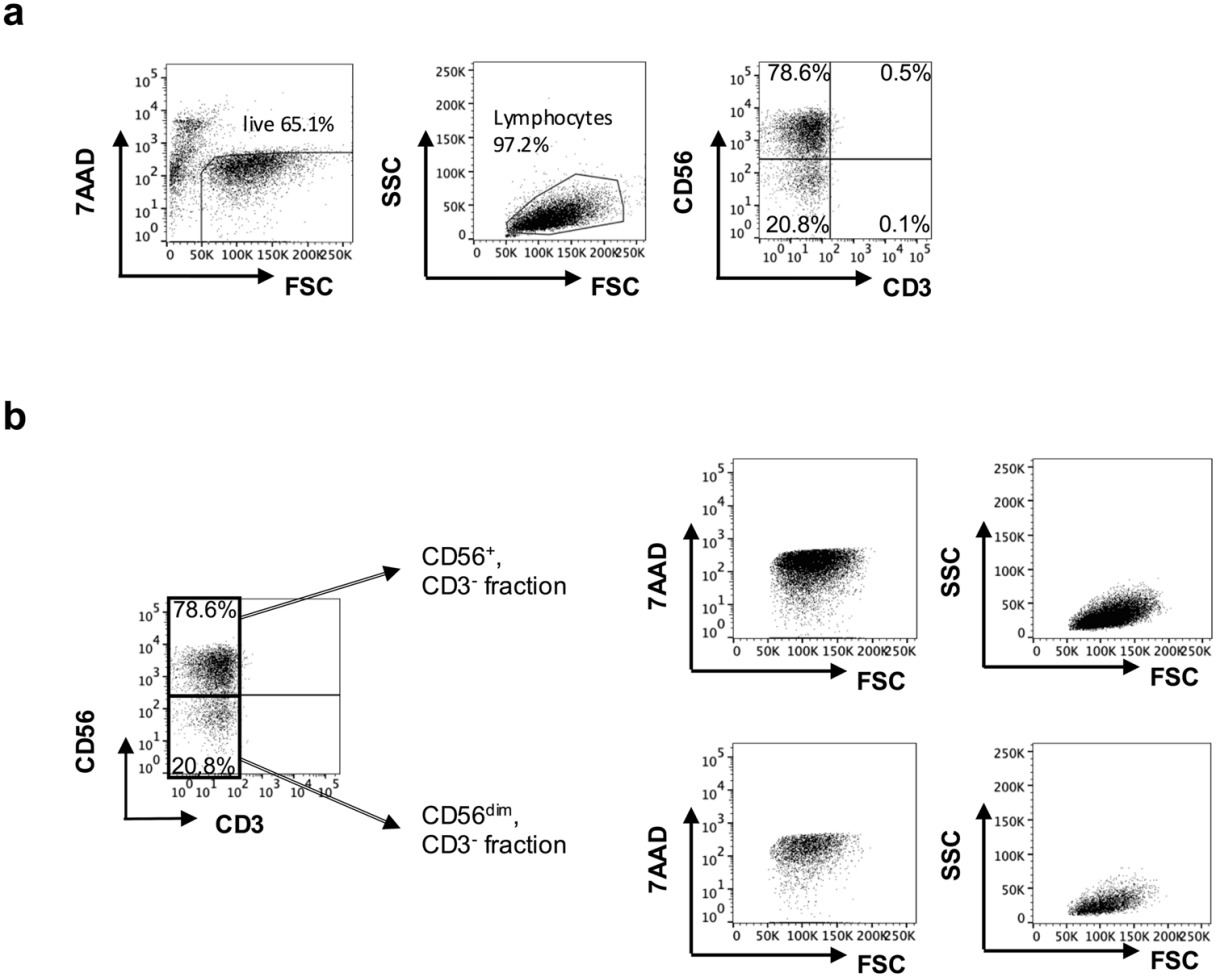
The phenotype of eNK cells fraction the phenotypic characteristics of the eNK cells were analyzed by using flow cytometry. **a** eNK cells were stained with anti-CD3 mAb, anti-CD56mAb, and 7AAD. Representative flow panels show the percentages of CD3^−^CD56^+^ NK cells, CD3^+^CD56^+^ NKT cells, and CD3^+^CD56^−^ T cells among the live lymphocyte population. **b** 7AAD/FSC and SSC/FSC properties were used to distinguish CD56^+^CD3^−^ and CD56^dim^CD3^−^ NK cell fractions in eNK cells

NKT (CD56^+^, CD3^+^) and T cells (CD56^−^, CD3^+^) were virtually absent (Fig. 1a). Both CD56^+^ and CD56^dim^ cells were relatively large lymphocytes that did not stain with 7-AAD, indicating that the viability of both fractions was well conserved (Fig. 1).

**Fig. 2:**
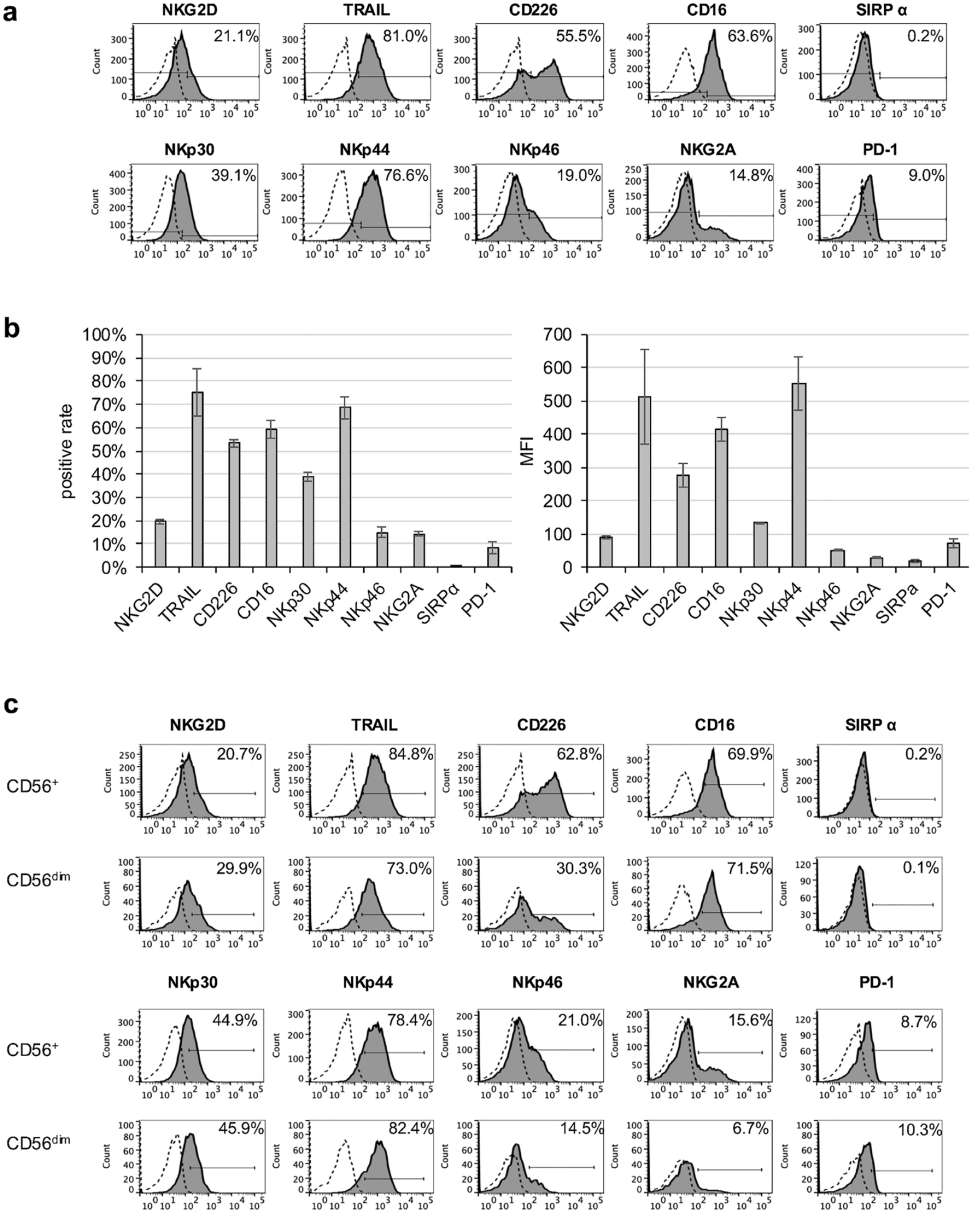
Expression of functional molecules in eNK cells. Functional molecules (NKG2D, TRAIL, CD226, CD16, NKp30, NKp44, NKp46, NKG2A, SIRPα, and PD-1) on whole eNK cells were evaluated using flow cytometry. **a** Representative histograms are shown. The filled histograms indicate those expressions, whereas the dotted open histograms indicate negative staining with isotype-matched control mAb. **b** The bar graph shows the average of each marker expressed by eNK cells (left: % of positive, right: MFI). Data are expressed as mean ± SEM (n = 3). (c) Representative histograms for functional molecules of each subset are shown in CD56^+^CD3^−^ eNK cells (upper) and CD56^dim^CD3.^−^ eNK cells (lower). The expressions of each molecule (gray-shaded) are shown with comparisons against isotype controls (dotted line)

The expression of major functional molecules characteristic of NK cells (NKG2D, TRAIL, CD226, CD16, NKp30, NKp44, NKp46, NKG2A, SIRPα, and PD-1) was also analyzed on eNK cells via flow cytometry. The eNK cells unimodally expressed NKG2D, TRAIL, CD16, NKp30, and NKp44, whereas CD226 and NKp46 were bimodally expressed (Fig. 2a). eNK cells showed the high expression of TRAIL, CD226, and CD16 (Fig. 2b, Table Sup.1).

CD56^+^ and CD56^dim^ cells shared a similar expression pattern for these surface molecules and showed no differences in expression levels (Fig. 2c). These data indicate that eNK cells have a highly antitumor phenotype.

### eNK cells have a high capacity for the production of intracellular cytotoxic factors and cytokines

**Fig. 3:**
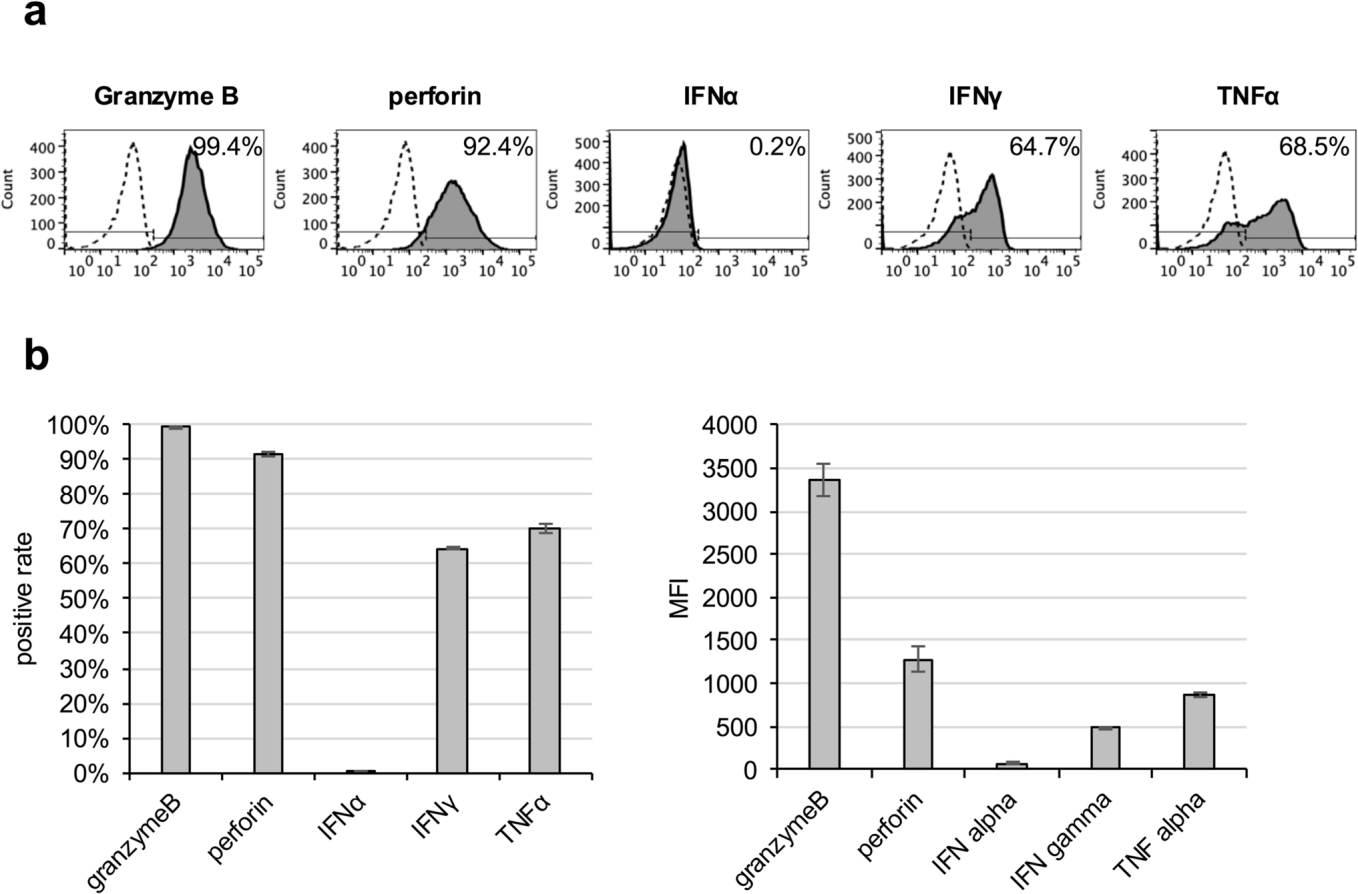
Expression of representative intracellular cytotoxic factors and cytokines in eNK cells. The expressions of granzyme B, perforin, IFNα, IFNγ, and TNFα on whole eNK cells were evaluated using intracellular flow cytometry. **a** Representative histograms are shown. The filled histograms indicate those expressions, whereas the dotted open histograms indicate negative staining with isotype-matched control mAb. **b** The bar graph shows the average of each marker expressed by eNK cells (left: % of positive, right: MFI). Data are expressed as mean ± SEM (n = 3)

Intracellular cytotoxic substances and cytokines are important factors in the antitumor activity of NK cells. We evaluated the expression of typical cytotoxic substances (granzyme B and perforin) and cytokines (TNFα, IFNα, and IFNγ) in eNK cells via intracellular flow cytometry. Granzyme B, perforin, IFNγ, and TNFα were highly expressed (Fig. 3, Table Sup. 2). These data indicate that eNK cells have high cytotoxic activity potential through cytolytic granule release and cytokine secretion.

### Expression of ligands for antitumor molecules on natural killer cells differs among human liver cancer cell lines

The binding of antitumor and immune checkpoint molecules to their ligands is an essential component of the therapeutic efficacy of immune cell-based therapy and is associated with various phenotypes [25]. Hence, sensitivity to NK cells may differ depending on the cancer cell phenotype. In previous studies, we reported that TRAIL expressed on NK cells is an important molecule in immunotherapy against HCC [13, 14]. Additionally, NKG2D and CD226 expressed on NK cells have been shown to play key roles in antitumor activity against HCC [26, 27]. Furthermore, anti-PD-L1 mAb are used in HCC patients, and anti-CD47 mAb have also been reported as promising therapeutic strategies for HCC [28]. Therefore, these are the rationale for analyzing NKG2D, TRAIL, CD226, ligands, PD-L1,2, and CD47 in this study. These molecules were analyzed in eight different liver cancer cell lines (HepG2, C3A, HuH7, PLC/PRF/5, SNU-387, SNU-423, SNU-449, and SK-HEP-1).

**Fig. 4:**
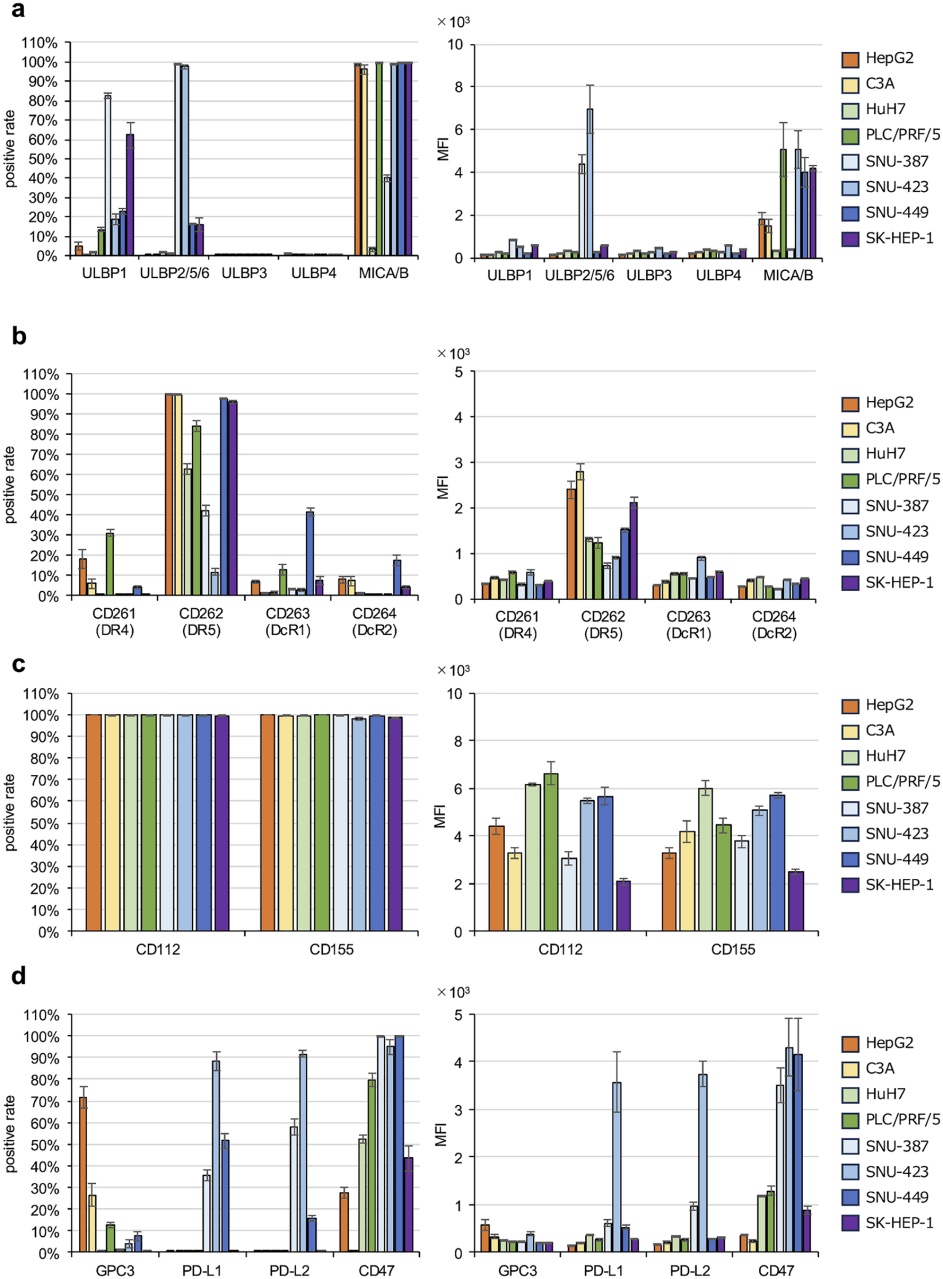
Expression of NKG2D ligands, TRAIL receptors, CD226 ligands, GPC3, PD-L1, PD-L2, and CD47 in liver cancer cell lines. Expression of **a** NKG2D ligands (ULBP1 ~ 6 and MICA/B), **b** TRAIL receptors (DR4, DR5, DcR1, and DcR2), **c** CD226 ligands (CD112 and CD155), and **d** GPC3, PD-L1, PD-L2, and CD47 were analyzed on HCC cell lines using flow cytometry. The percentage of positive cells and MFI were calculated using FlowJo. All data are expressed as the mean ± SEM (*n* = 3). Each graph shows the following cell lines (from left to right): HepG2, C3A, HuH7, PLC/PRF/5, SNU-387, SNU-423, SNU-449, and SK-HEP-1

Of the NKG2D ligands, consisting of ULBP1–6 and MICA/B, ULBP1 was expressed at different frequencies on different cell lines, but the expression intensity was low, regardless of the expression frequency (Fig. 4a, Table Sup.3a). ULBP3 and ULBP4 were not expressed on any of the cell lines (Fig. 4a). MICA/B was highly expressed, except on HuH7 cells, but its MFI differed depending on the cell line (Fig. 4a, Table Sup.3a). Thus, all ligands for NKG2D were barely expressed on HuH7 cells. These data indicate that liver cancer cell lines, other than HuH7, are likely to be sensitive to NKG2D, albeit with varying expression levels.

TRAIL receptors expressed on the cell surface can be divided into death (DR4 and DR5) and decoy receptors (DcR1 and DcR2). DR5 (CD262), the major TRAIL death receptor, was expressed on the cell surface of almost 100% of HepG2, C3A, SNU-449, and SK-HEP-1 cells, but only in approximately 10% of SNU423 cells (Fig. 4b, Table Sup.3b). Decoy receptor expression was low in most cell lines. These data suggest that liver cancer cell lines are susceptible to varying degrees of TRAIL cytotoxicity, and that evasion of such cytotoxicity by decoy receptors is unlikely to occur.

The binding of CD226 to CD112 or CD155 is known to result in NK cell effector function. CD112 and CD155 were expressed to varying degrees on almost 100% of the cell lines (Fig. 4c, Table Sup.3c). These data indicate that liver cancer cell lines may induce activation signals in NK cells via CD226 to varying degrees.

We also evaluated the expression of GPC3, an HCC-specific marker. GPC3 expression was high in HepG2 cells and moderate in C3A cells (Fig. 4d). However, the other cell lines showed poor GPC3 expression. PD-L1 and PD-L2 expression was observed in SNU-387, SNU-423, and SNU-449 cells but not in the other cell lines (Fig. 4d, Table Sup.3d). Expression of CD47, a ligand for SIRPα, varied widely among the cell lines (Fig. 4d, Table Sup.3d). These results suggest that the impact of these two signaling pathways on immune regulatory mechanisms varies widely among the cell lines. Based on the findings, we selected HepG2, HuH7, and SNU-423 as targets for cytotoxic assays, as they exhibited distinct expression patterns of key molecules targeted by NK cells, including TRAIL-R, PD-L1, and NKG2D ligands.

### eNK cells exhibited enhanced antitumor activity when co-cultured with HCC cells

To examine whether eNK cells are further activated by HCC cell lines, we conducted experiments where eNK cells were co-cultured with HCC cell lines (HepG2, HuH7, and SNU-423) for 4 h without further stimulation. We then observed the expression of CD107a on the cell surface, as well as IFN-γ and TNFα within the cytoplasm of eNK cells. In all three cell lines, particularly HepG2 and HuH7, co-culture significantly increased CD107a expression on the eNK cell surface (Fig. 5). Similarly, IFN-γ and TNFα expressions in the cytoplasm of eNK cells were also upregulated following co-culture with the HCC cell lines (Fig. 5).

**Fig . 5:**
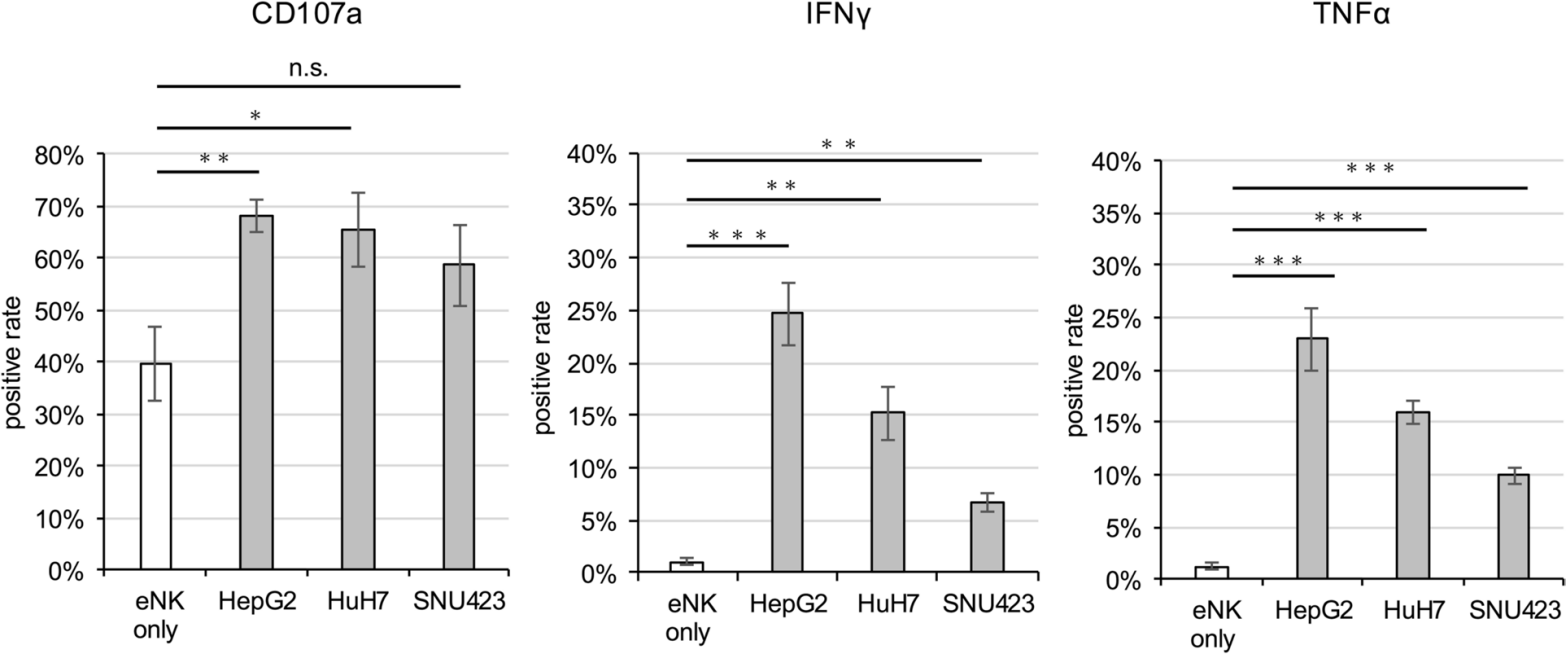
Expression of CD107a, TNFα, and IFNγ on eNK cells after co-cultured with HCC cell lines. The expression of CD107a, TNFα, and IFNγ on eNK cells were evaluated after co-cultured with HepG2, HuH7, and SNU-423 cells for 4 h. Only eNK cells were cultured as the control group. The bar graph indicates the average positive rate of each marker expressed on lymphocyte-gated and CD56^+^ cells. All data are expressed as the mean ± SEM (*n* = 3). Significance differences are indicated as *p < 0.05, **p < 0.01, and ***p < 0.001.

### eNK cells exhibit high cytotoxicity against hepatocellular carcinoma cell lines

**Fig. 6:**
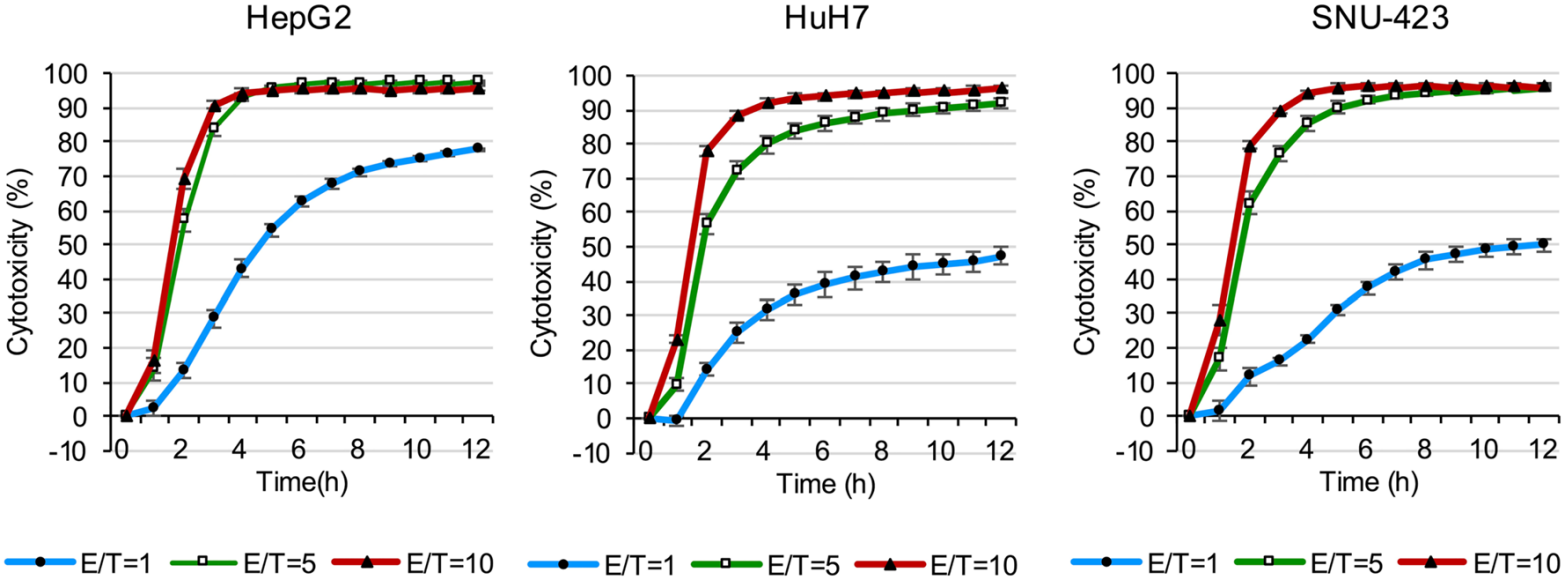
Cytotoxic effects of eNK cells in hepatocellular carcinoma cells. HepG2, HuH7, and SNU-423 cells were co-cultured with eNK cells at three different effector/target (E/T) ratios (E/T = 1, 5, and 10) for 12 h. The cytotoxic activity of eNK against hepatocellular carcinoma cells was evaluated using the xCELLigence software. All data are expressed as the mean ± SEM of three independent experiments. ● E/T = 1; □ E/T = 5; ▲ E/T = 10.

To investigate the antitumor effects of eNK cells, we continuously observed cytotoxicity using a real-time cell analyzer. When the E/T ratio was 10, eNK cells showed over 90% cytotoxic activity in all three cell types (Fig. 6). No significant difference was observed between when the E/T values were 5 and 10 (Fig. 6). However, a difference in cytotoxic activity was observed between the cell lines at an E/T ratio = 1. To compare the antitumor effects of eNK cells, we evaluate cytotoxicity against HCC cell lines (HepG2, HuH7, and SNU423) of NK cells isolated from peripheral blood (PBNK cells) derived three healthy volunteers. The results confirmed that the antitumor activity of eNK cells was significantly higher compared to that of PBNK cells (Fig.Sup.3). These results indicate that eNK cells have high antitumor activity in HCC and that their cytotoxicity can be influenced by the differential expression of ligands for NK cell antitumor molecules.

### Perforin, granzyme B, and TRAIL are highly related to the cytotoxicity of eNK cells

**Fig. 7:**
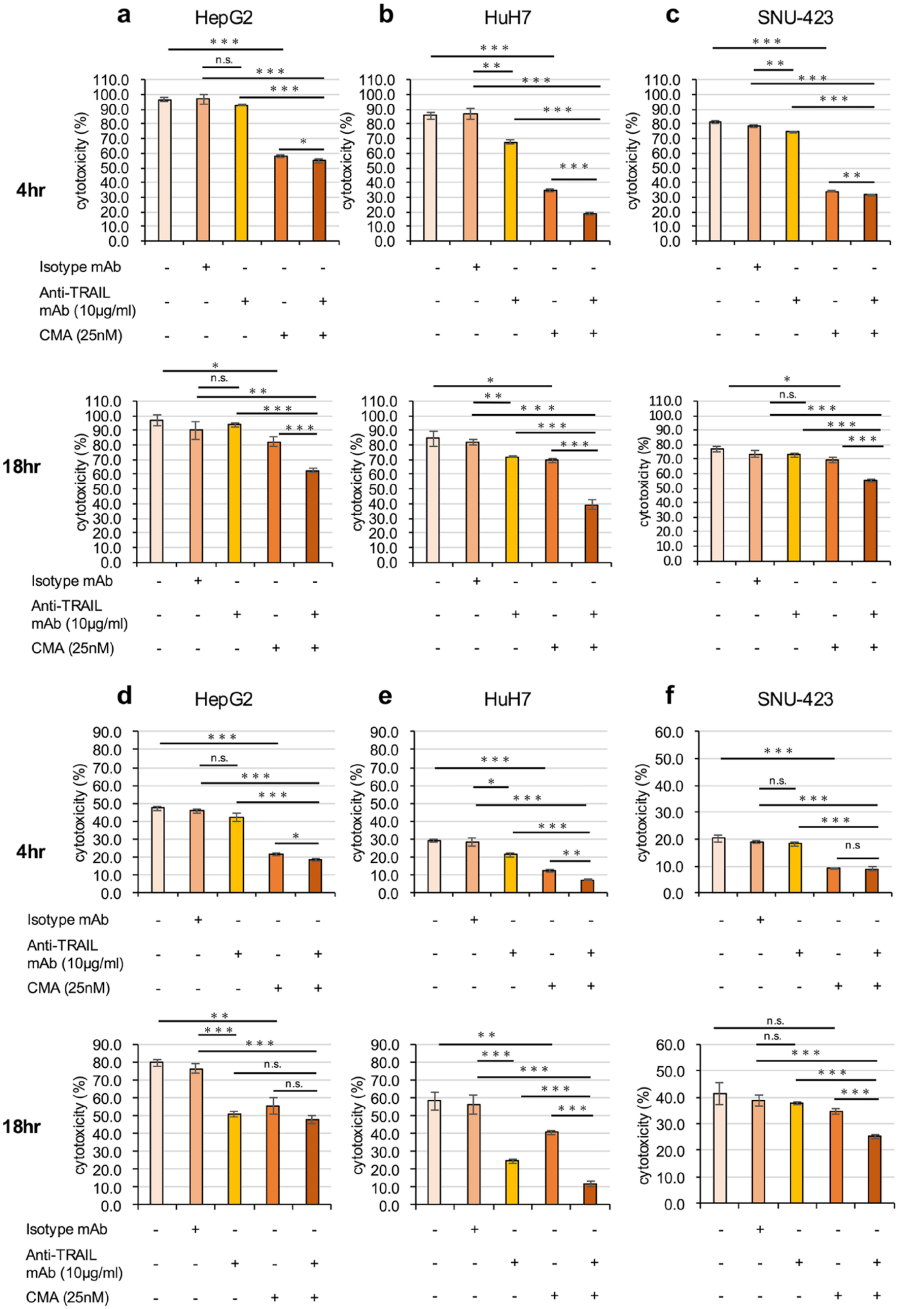
Mechanisms of the cytotoxic effects of eNK cells in hepatocellular carcinoma cells. HepG2, HuH7, and SNU-423 cells were co-cultured with eNK cells at effector/target (E/T) = 10 (**a**–**c**), E/T = 1 (**d**–**f**) for 4 (upper) and 18 h (lower) in the presence of 50 ng/mL IL-15, 10 μg/mL anti-TRAIL mAb, and/or 25 nmol/L concanamycin A (CMA). The cytotoxic activity of eNK cells in hepatocellular carcinoma cells was evaluated using a.^51^Cr-release assay. All data are expressed as the mean ± SEM of four replicates. Significance differences are indicated as *p < 0.05, **p < 0.01, and ***p < 0.001

To investigate the mechanisms underlying the observed cytotoxicity of eNK cells, we examined the contribution of TRAIL and perforin/granzyme B, which are highly expressed on eNK cells. Cytotoxicity was significantly inhibited in all three cell lines in the presence of CMA (Fig. 7).

When cultured at an E/T ratio of 10, anti-TRAIL mAb suppressed the cytotoxicity of eNK cells targeting HepG2 cells in the presence of CMA, but did not suppress it when used alone (Fig. 7a). However, at an E/T ratio = 1, the anti-TRAIL mAb alone inhibited cytotoxicity 18 h after the addition of eNK cells (Fig. 7d). In addition, comparing the presence or absence of anti-TRAIL mAb in the presence of CMA at an E/T ratio = 1, cytotoxicity tended to be lower in the former group, but not significantly (Fig. 7d). Cell damage of HuH7 cells was inhibited in the presence of CMA and/or anti-TRAIL mAb 4 and 18 h after the addition of eNK cells (Fig. 7b, e). At an E/T ratio = 10, the anti-TRAIL mAb alone slightly suppressed cell injury against SNU-423 after 4 h, whereas no significant difference was observed at 18 h (Fig. 7c). At an E/T ratio = 1, there was no significant difference observed between treatment with the anti-TRAIL mAb alone or with CMA alone at 18 h, but inhibition was observed in the presence of both (Fig. 7f). These data suggest that TRAIL and perforin/granzyme B pathways are closely involved in the cytotoxicity of eNK cells against all three HCC cell lines.

## Discussion

NK cells have four antitumor pathways: activated receptors/antitumor molecules, the antibody-dependent cellular cytotoxicity (ADCC) effect, cytolytic granule release, and cytokine secretion [2]. Various cell surface and intracellular factors are involved in these four pathways, and their activation induces cell injury through distinct signaling mechanisms. The key activated receptors and antitumor molecules include NKp30, NKp44, NKp46, TRAIL, CD226, and NKG2D. Among these, NKp30, NKp44, and NKp46 are classified as natural cytotoxic receptors. These receptors regulate cytotoxic activity and cytokine-secreting through downstream signal activation following binding with FcεRIγ and/or CD3ζ (NKp46 and NKp30) and DAP12 (NKp44) after binding to their ligands [29, 30]. After TRAIL binds to its death receptors (DR4 and DR5), they activate the extrinsic and intrinsic apoptosis pathways and induce transcriptional events leading to NF-κB-dependent proinflammatory cytokine expression [31, 32]. CD226 activates downstream signaling cascades that activate phosphatidylinositol-4,5-bisphosphate phosphodiesterase gamma-2 (PLCγ2), ERK, and AKT downstream and remove the negative regulator of NK cell activation through phosphorylation of the forkhead box protein O1 transcription factor via activated AKT [27, 33]. NKG2D binding with its ligand promotes cytotoxicity, granule release, and cytokine release through activation of the DAP10 signaling molecule and the following signals: PLCγ2, c-Jun-NH (2)-terminal kinase, phosphatidylinositol 3-hydroxy kinase (PI3K), and Janus kinase 2-signal transducer and activator of transcription 5 (JAK-STAT5) pathway [26, 34]. CD16 (IgG-activated Fc receptor III) recruits SYK family kinases via crosslinking by immune complexes and induces ADCC effects by activating several other signaling molecules and their downstream signals, including the PI3K and SOS pathways [35].

NK cells secrete cytolytic granules, including the pore-forming protein, perforin, and the serine protease, granzyme B, which synergistically mediate the apoptosis of target cells[36]. NK cells also secrete various cytokines, such as TNFα, IFNα, and IFNγ. TNFα induces apoptosis and necroptosis through the kinase receptor-interacting serine/threonine-protein kinase 1 after binding to TNFR1, which is associated with the death domain (TRADD) [37, 38]. IFNα and IFNγ can bind to their respective receptors and activate several pathways, including the JAK-STAT pathway, to coordinate different cell functions, such as immune regulation, leukocyte transportation, cell proliferation, apoptosis, and antimicrobial, antitumor, and pro-tumor effects [39, 40].

We analyzed these cell surface and intracellular cytotoxic factors expressed on eNK cells. Antitumor-related molecules (TRAIL, CD226, and NKG2D), ADCC-inducing molecule (CD16) and intracellular cytotoxic substances (perforin, granzyme B, TNFα, and IFNγ) were found to be highly expressed. The eNK cells were derived from genetically modified iPSCs through differentiation into hematopoietic progenitor cells (HPCs) and subsequently into NK cells. Generally, during the maturation process from HPCs to NK cells, the expression of surface molecules fluctuates, and their functions vary depending on the stage of maturation [41–43]. The high expression of factors involved in all four antitumor pathways of NK cells indicates that eNK cells exhibit a robust capacity and possess the phenotype of mature NK cells.

In the activated receptor/antitumor molecular pathway, the expression of their ligands on cancer cells is necessary for efficient binding to the receptor and an effective immune response through NK cell activation. Cancer cell lines are widely used in in vitro studies. Although HepG2 and HuH7 are the most commonly used cell lines in hepatocarcinoma studies, approximately 40 liver cancer cell lines have been established from patients with different disease backgrounds [44]. We initially selected eight cell lines (HepG2, C3A, HuH7, PLC/PRF/5, SNU-387, SNU-423, SNU-449, and SK-HEP-1). SK-HEP-1 was derived from a patient with adenocarcinoma, whereas the other cell lines were derived from patients with HCC. Additionally, each cell line contained different mutated genes [44]. Although many studies using these cell lines have been conducted, the expression status of ligands for NK cell antitumor molecules in liver cancer cell lines has not yet been elucidated. In this study, we focused on the antitumor molecules (TRAIL, CD226, and NKG2D) that trigger different antitumor signals, and examined the expression status of these ligands in eight liver cancer cell lines. Our results revealed that each cell line could be characterized according to its sensitivity to TRAIL, NKG2D, CD47, and PD-1. HuH7 barely expressed NKG2D ligands, whereas almost all other cell lines appeared to be sensitive to NKG2D. The expression of DR5 varied between approximately 10% and 100%. Furthermore, each cell line showed variable expression of CD47, PD-L1, and PD-L2. Even in patients, HCC shows heterogeneous features at both the molecular and morphological levels. Thus, the key to enhance the treatment efficacy of eNK cells is whether they can show antitumor effects against cancer cells with various phenotypes. We selected HepG2 (high sensitivity to NKG2D and TRAIL, low sensitivity to PD-1), HuH7 (moderate sensitivity to TRAIL, low sensitivity to NKG2D and PD-1), and SNU423 (low sensitivity to TRAIL, high sensitivity to NKG2D and PD-1) cells for cytotoxicity assays. The eNK cells showed remarkably high cytotoxicity against all three cell lines. We did not observe a significant difference in the cytotoxicity of eNK cells between the cell lines at E/T ratios = 5 and 10; however, a difference was observed in the cytotoxic activity between cells at an E/T ratio = 1. This may be due to differences in the expression of ligands for the antitumor molecules of NK cells on the surfaces of each HCC cell line.

It has been reported that, in the process of serial killing, NK cells initially predominantly use the perforin/granzyme B pathway to rapidly kill tumor cells; however, they later switch to death receptor-mediated cytotoxicity, which requires a longer time to induce cell death when granules are reduced [45]. Our blocking assays and CD107a degranulation assay revealed that the TRAIL and perforin/granzyme B pathways are largely involved in the cytotoxicity mechanisms of eNK cells, and the effects of perforin/granzyme B and TRAIL pathway inhibition were consistent with previous reports [45]. Thus, eNK cells also have the potential to switch killing mechanisms during the serial killing.

TGF-β signaling has been proven to suppress NK cell activity within the tumor microenvironment (TME), which contributes to the overall immune evasion by HCC tumors [46]. This suppression is part of the broader effect of TGF-β in promoting an immunosuppressive environment, making NK cells less effective in their role against tumor cells. To address this issue, for example, knocking down the TGF-β receptor 2 gene in eNK cells may help overcome the immunosuppressive TME and enhance therapeutic efficacy. Similarly, in AML patients, where high levels of TGF-β1 are expressed in the bone marrow, resistance to NK cell-based immunotherapy is a concern [47]. However, genetic manipulation of eNK cells to target the TGF-β receptor 2 gene could potentially improve the effectiveness of these therapies.

NK-92 cells are a well-established and extensively characterized human NK cell line that has demonstrated promising anticancer activity against various cancer types in clinical trials [48, 49]. In the context of HCC, a previous study reported the generation of GPC3-specific CAR-engineered NK cells using NK-92 cells, which showed efficacy in HCC treatment [16]. In this study, NK-92 cells were used as one of the indicators for evaluating cell sources in immunotherapy. They were assessed using the same analytical system employed to evaluate the antitumor effects of eNK cells. Flow cytometry and cytotoxicity assays revealed that NK-92 cells exhibited higher expression levels of intracellular cytotoxic substances and cytokines (Fig. Sup.1c,d, Table Sup.2). However, their expression of TRAIL, CD226, and CD16 were comparatively lower (Fig. Sup.1a,b, Table Sup.1). Furthermore, NK-92 cells displayed greater cytotoxicity than PBNK cells (Fig. Sup.2,3), but their cytotoxic activities were generally lower than those observed in eNK cells.

NK-92 cells are easy to culture and expand in vitro, making them a promising platform for adoptive cell therapy. Furthermore, they can be genetically engineered to express specific receptors or cytokines, thereby enhancing their efficacy against resistant tumors [48, 49]. However, NK-92-based therapies have certain limitations. A significant drawback is that, because NK-92 cells originate from a malignant lymphoma source, they must be irradiated before clinical use to prevent potential tumorigenicity, restricting their ability to proliferate and persist in vivo [50, 51].

From the perspective of achieving efficient proliferation in vitro while addressing the limitations of in vivo proliferation, differentiating NK cells from iPSCs for use in immunotherapy appears to be a rational approach. The hiPSCs from which the eNK cells are derived have been thoroughly evaluated for their limited proliferation ability following differentiation induction and their non-tumorigenicity, making them sufficiently reliable for clinical application [21]. Since eNK cells are not subjected to immortalization processes, they lack unlimited proliferative potential, thereby reducing the risk of uncontrolled proliferation or other adverse effects in vivo. This represents a key safety advantage when considering their therapeutic application. Furthermore, we have successfully established universal donor iPSCs with MHC class I/II knockout from these hiPSCs [52]. If eNK cells can be derived from these universal donor iPSCs in the future, they are expected to serve as a safer and more effective source for cell therapy, with fewer rejection reactions and enhanced safety. Research toward this goal is currently ongoing.

In conclusion, eNK cells have a strong antitumor phenotype and high cytotoxic activity against HCC cell lines with various phenotypes. Therefore, eNK cells may represent a novel therapeutic strategy candidate for HCC. Although we focused on the in vitro cytotoxicity and mechanisms of action of eNK cells in the present study, their effects in in vivo models remain unclear and require further investigation.

## Supplementary Information

Below is the link to the electronic supplementary material.

Supplementary file1 (DOCX 6297 KB)

## Data Availability

No datasets were generated or analyzed during the current study.

## Abbreviations

7-AAD: 7-Amino-actinomycin D
ADCC: Antibody-dependent cellular cytotoxicity
CAR: Chimeric antigen receptor
CMA: Concanamycin A
E/T: Effector/target
GPC3: Glypican 3
HCC: Hepatocellular carcinoma
HPC: Hematopoietic progenitor cell
IFN: Interferon
iPSC: Induced pluripotent stem cell
JAK-STAT: Janus kinase-signal transducer and activator of transcription
mAb: Monoclonal antibodies
NK: Natural killer
PBMC: Peripheral blood mononuclear cell
PBNK: Peripheral blood natural killer
PD-1: Programmed death 1
PI3K: Phosphatidylinositol 3-hydroxy kinase
PLCγ2: Phosphatidylinositol-4,5-bisphosphate phosphodiesterase gamma-2
SIRPα: Signal regulatory protein-α
TNF: Tumor necrosis factor
TRAIL: Tumor necrosis factor-related apoptosis-inducing ligand
