# Supplementary material for: Antitumor effects of natural killer cells derived from gene-engineered human-induced pluripotent stem cells on hepatocellular carcinoma
Source: Cancer Immunol Immunother. 2025 Feb 4;74(3):99. doi: 10.1007/s00262-025-03940-5 (PMC11794780; doi:10.1007/s00262-025-03940-5)
Supplement: Supplementary file 1 — Supplementary file1 (DOCX 6297 KB) [file 262_2025_3940_MOESM1_ESM.docx]

**Supplementary Material**

## Supplementary Figures


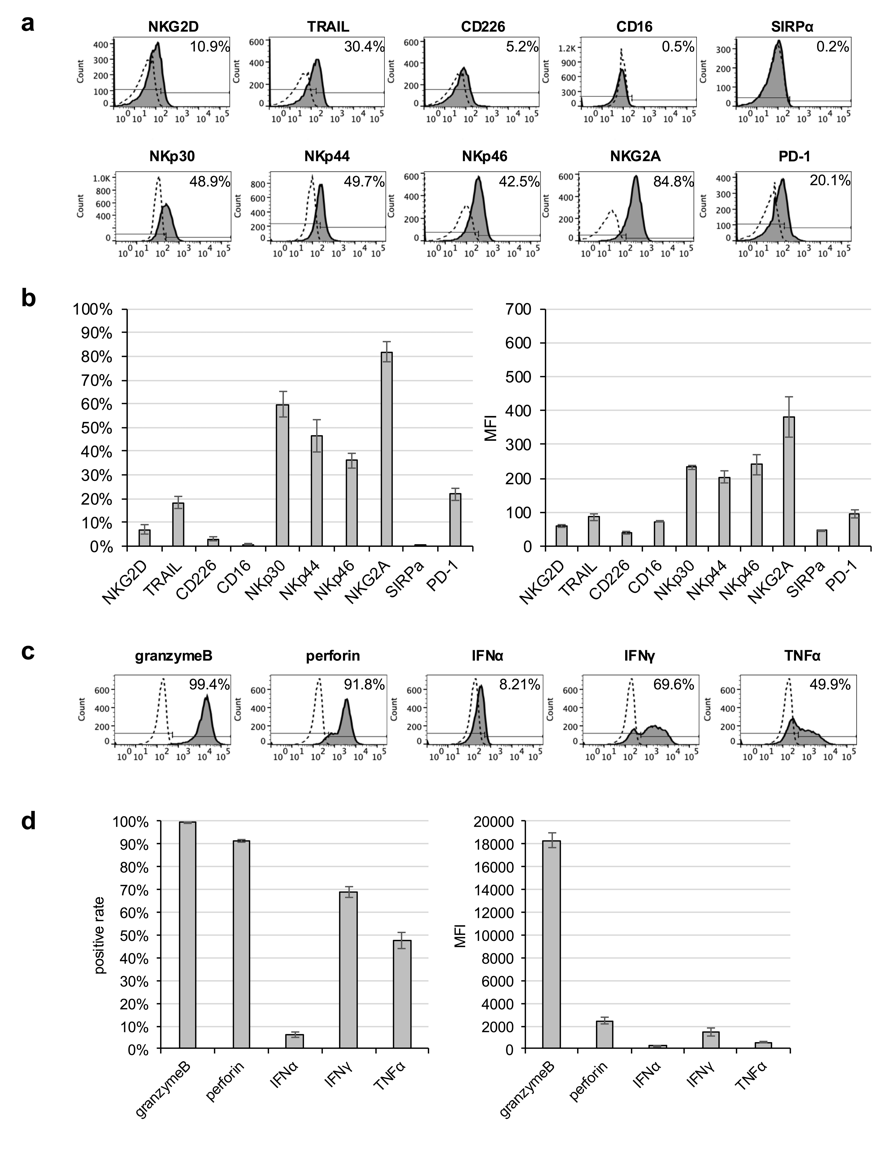


**Supplementary Figure 1**. **Expression of representative functional molecules of natural killer cells and intracellular cytotoxic factors and cytokines in NK-92 cells.**

Functional molecules (NKG2D, TRAIL, CD226, CD16, NKp30, NKp44, NKp46, NKG2A, SIRPα, and PD-1) on whole NK-92 cells were evaluated using flow cytometry. (a) Representative histograms are shown. The filled histograms indicate those expressions, whereas the dotted open histograms indicate negative staining with isotype-matched control mAb. (b) The bar graph shows the average of each marker expressed by NK-92 cells (left: % of positive, right: MFI). Data are expressed as mean ± SEM (n = 3). The expressions of granzyme B, perforin, IFNα, IFNγ, and TNFα on whole NK-92 cells were evaluated using intracellular flow cytometry. (c) Representative histograms are shown. The filled histograms indicate those expressions, whereas the dotted open histograms indicate negative staining with isotype-matched control mAb. (d) The bar graph shows the average of each marker expressed by NK-92 cells (left: % of positive, right: MFI). Data are expressed as mean ± SEM (n = 3).

.


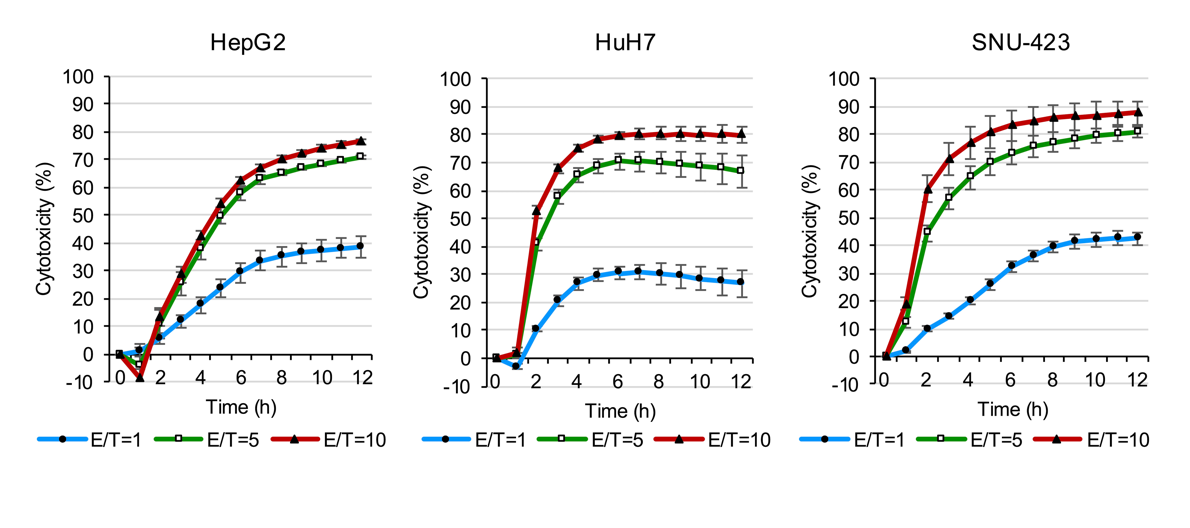


**Supplementary Figure 2**. **Cytotoxic effects of NK-92 cells in hepatocellular carcinoma cells.**

HepG2, HuH7, and SNU-423 cells were co-cultured with NK-92 cells at three different effector/target (E/T) ratios (E/T = 1, 5, and 10) for 12 h. The cytotoxic activity of NK-92 against hepatocellular carcinoma cells was evaluated using the xCELLigence software. All data are expressed as the mean ± SEM of three independent experiments. ● E/T = 1; □ E/T = 5; ▲ E/T = 10.


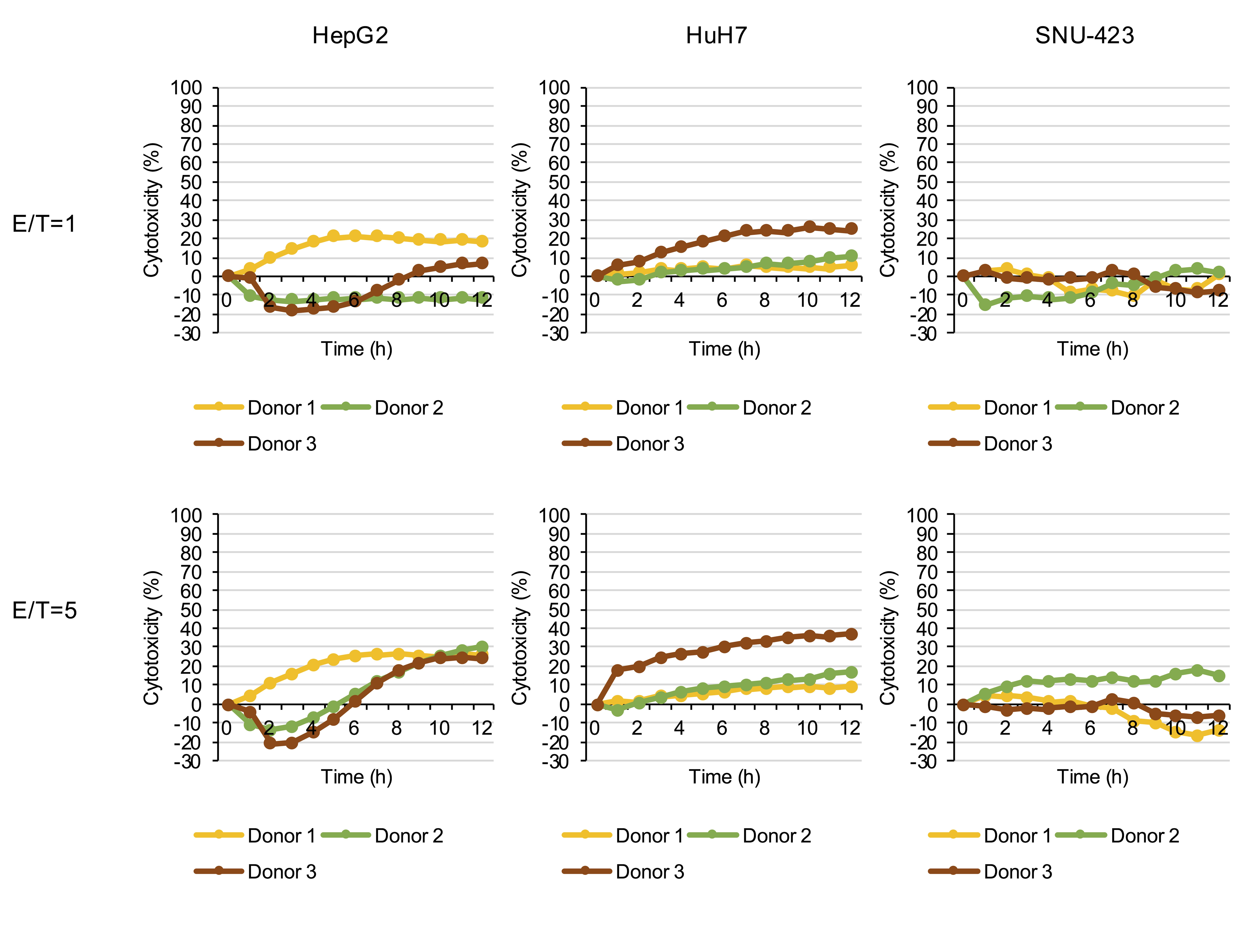


**Supplementary Figure 3. Cytotoxic effects of PBNK cells in hepatocellular carcinoma cells.**

HepG2, HuH7, and SNU-423 cells were co-cultured with PBNK cells from three different donors at two different effector/target (E/T) ratios (E/T = 1 and 5) for 12 h. The cytotoxic activity of PBNK cells against hepatocellular carcinoma cells was evaluated using the xCELLigence software.

| **Molecule** | **eNK** | | | | | | | | | **NK-92** | | | | | | | | |
| --- | --- | --- | --- | --- | --- | --- | --- | --- | --- | --- | --- | --- | --- | --- | --- | --- | --- | --- |
|  | **Positive rate**  (%, mean ± SEM) | | | **MFI**  (mean ± SEM) | | | | | | **Positive rate**  (%, mean ± SEM) | | | **MFI**  (mean ± SEM) | | | | | |
| CD56 | 77.3 | ± | 1.9 | 1193.9 | ± | 222.1 | (22.7 | ± | 1.1) | 99.9 | ± | 0.0 | 6329.6 | ± | 591.3 | (73.8 | ± | 1.4) |
| CD3 | 0.9 | ± | 0.2 | 12.5 | ± | 2.8 | (7.3 | ± | 1.4) | 0.5 | ± | 0.1 | 27.7 | ± | 3.3 | (11.5 | ± | 0.4) |
| NKG2D | 19.5 | ± | 1.2 | 89.1 | ± | 4.2 | (16.5 | ± | 0.4) | 8.4 | ± | 2.4 | 60.5 | ± | 4.8 | (16.4 | ± | 0.2) |
| TRAIL | 75.0 | ± | 10.0 | 512.3 | ± | 141.8 | (16.5 | ± | 0.4) | 20.7 | ± | 4.9 | 85.3 | ± | 9.4 | (16.4 | ± | 0.2) |
| CD226 | 53.3 | ± | 1.6 | 276.3 | ± | 34.1 | (16.5 | ± | 0.4) | 3.6 | ± | 1.2 | 38.7 | ± | 4.0 | (16.4 | ± | 0.2) |
| CD16 | 59.1 | ± | 4.0 | 412.8 | ± | 36.1 | (25.5 | ± | 0.5) | 0.7 | ± | 0.3 | 72.9 | ± | 2.9 | (76.6 | ± | 4.3) |
| NKp30 | 38.6 | ± | 1.8 | 132.6 | ± | 3.0 | (25.2 | ± | 0.4) | 59.6 | ± | 5.4 | 232.2 | ± | 6.1 | (75.7 | ± | 9.9) |
| NKp44 | 68.4 | ± | 4.7 | 553.6 | ± | 80.1 | (25.4 | ± | 0.5) | 46.6 | ± | 6.7 | 204.1 | ± | 16.6 | (70.4 | ± | 5.2) |
| NKp46 | 14.9 | ± | 2.3 | 51.0 | ± | 2.6 | (14.4 | ± | 1.0) | 36.2 | ± | 3.2 | 241.2 | ± | 30.5 | (30.4 | ± | 6.1) |
| NKG2A | 14.4 | ± | 1.0 | 28.9 | ± | 0.5 | (7.5 | ± | 0.2) | 81.9 | ± | 4.3 | 380.3 | ± | 59.7 | (9.6 | ± | 0.5) |
| SIRPα | 0.3 | ± | 0.2 | 20.8 | ± | 3.5 | (13.3 | ± | 1.2) | 0.2 | ± | 0.0 | 45.8 | ± | 0.8 | (34.1 | ± | 2.6) |
| PD-1 | 8.6 | ± | 2.7 | 73.0 | ± | 13.1 | (16.6 | ± | 2.1) | 21.9 | ± | 2.5 | 93.9 | ± | 11.4 | (17.9 | ± | 0.2) |

**Supplementary Table 1.** Positive rate and MFI for each functional molecule in eNK and NK-92 cells. MFI for the isotype control is shown in parentheses.

**Supplementary Table 2.** Positive rate and MFI for each intracellular cytotoxic factor and cytokine in eNK and NK-92 cells.

| **Molecule** | **eNK** | | | | | | **NK-92** | | | | | |
| --- | --- | --- | --- | --- | --- | --- | --- | --- | --- | --- | --- | --- |
|  | **Positive rate**  (%, mean ± SEM) | | | **MFI**  (mean ± SEM) | | | **Positive rate**  (%, mean ± SEM) | | | **MFI**  (mean ± SEM) | | |
| Isotype control | 0.0 | ± | 0.0 | 51.30 | ± | 1.2 | 0.0 | ± | 0.0 | 162.7 | ± | 11.9 |
| Granzyme B | 99.2 | ± | 0.2 | 3354.00 | ± | 192.5 | 99.6 | ± | 0.1 | 18296.7 | ± | 599.6 |
| Perforin | 91.6 | ± | 0.7 | 1274.00 | ± | 147.2 | 91.3 | ± | 0.7 | 2452.7 | ± | 280.6 |
| IFNα | 0.2 | ± | 0.0 | 77.17 | ± | 4.3 | 6.2 | ± | 1.4 | 306.3 | ± | 10.7 |
| IFNγ | 64.5 | ± | 0.1 | 500.67 | ± | 3.3 | 69.0 | ± | 2.3 | 1469.0 | ± | 360.1 |
| TNFα | 70.3 | ± | 1.1 | 878.67 | ± | 28.4 | 47.5 | ± | 3.3 | 538.0 | ± | 64.1 |

**Supplementary Table 3a.** Positive rate and MFI for NKG2D ligands in liver cancer cell lines.

| **Positive rate** | **ULBP1**  Mean ± SEM (%) | | | **ULBP2/5/6**  Mean ± SEM (%) | | | **ULBP3**  Mean ± SEM (%) | | | **ULBP4**  Mean ± SEM (%) | | | **MICA/B**  Mean ± SEM (%) | | |
| --- | --- | --- | --- | --- | --- | --- | --- | --- | --- | --- | --- | --- | --- | --- | --- |
| Cell line |  |  |  |  |  |  |  |  |  |  |  |  |  |  |  |
| **HepG2** | 4.93 | ± | 2.14 | 0.17 | ± | 0.01 | 0.17 | ± | 0.03 | 1.39 | ± | 0.21 | 98.83 | ± | 0.69 |
| **C3A** | 0.79 | ± | 0.10 | 0.19 | ± | 0.07 | 0.18 | ± | 0.06 | 0.28 | ± | 0.03 | 96.23 | ± | 2.48 |
| **HuH7** | 1.59 | ± | 0.44 | 1.76 | ± | 0.04 | 0.20 | ± | 0.03 | 0.26 | ± | 0.05 | 3.18 | ± | 1.13 |
| **PLC/PRF/5** | 13.50 | ± | 0.99 | 1.64 | ± | 0.09 | 0.10 | ± | 0.02 | 0.61 | ± | 0.08 | 99.70 | ± | 0.20 |
| **SNU-387** | 82.60 | ± | 1.23 | 98.70 | ± | 0.29 | 0.20 | ± | 0.02 | 0.20 | ± | 0.01 | 40.13 | ± | 0.42 |
| **SNU-423** | 18.70 | ± | 3.06 | 97.60 | ± | 0.92 | 0.28 | ± | 0.10 | 0.21 | ± | 0.05 | 99.00 | ± | 0.20 |
| **SNU-449** | 23.00 | ± | 1.39 | 16.47 | ± | 0.35 | 0.13 | ± | 0.02 | 0.65 | ± | 0.21 | 99.87 | ± | 0.03 |
| **SK-HEP-1** | 62.17 | ± | 6.54 | 15.97 | ± | 3.53 | 0.14 | ± | 0.01 | 0.73 | ± | 0.02 | 99.57 | ± | 0.09 |

| **MFI** | **ULBP1**  Mean ± SEM | | | **ULBP2/5/6**  Mean ± SEM | | | **ULBP3**  Mean ± SEM | | | **ULBP4**  Mean ± SEM | | | **MICA/B**  Mean ± SEM | | |
| --- | --- | --- | --- | --- | --- | --- | --- | --- | --- | --- | --- | --- | --- | --- | --- |
| Cell line | upper panel: respective molecules | | | | | | | | | | | | | | |
|  | lower panel: isotype control | | | | | | | | | | | | | | |
| **HepG2** | 176.00 | ± | 5.57 | 152.00 | ± | 7.00 | 152.00 | ± | 7.81 | 218.67 | ± | 7.62 | 1837.33 | ± | 286.38 |
|  | 114.67 | ± | 4.41 | 135.00 | ± | 3.51 | 135.00 | ± | 3.51 | 135.00 | ± | 3.51 | 114.67 | ± | 4.41 |
| **C3A** | 185.33 | ± | 11.32 | 192.67 | ± | 25.67 | 208.67 | ± | 29.76 | 258.67 | ± | 28.50 | 1471.33 | ± | 304.77 |
|  | 134.00 | ± | 8.96 | 180.33 | ± | 23.97 | 180.33 | ± | 23.97 | 180.33 | ± | 23.97 | 134.00 | ± | 8.96 |
| **HuH7** | 296.33 | ± | 1.20 | 371.33 | ± | 11.29 | 350.00 | ± | 24.52 | 389.33 | ± | 8.95 | 343.33 | ± | 9.21 |
|  | 234.00 | ± | 5.57 | 322.33 | ± | 4.63 | 322.33 | ± | 4.63 | 322.33 | ± | 4.63 | 234.00 | ± | 5.57 |
| **PLC/PRF/5** | 230.67 | ± | 10.73 | 250.67 | ± | 18.48 | 250.67 | ± | 13.86 | 360.33 | ± | 24.11 | 5095.00 | ± | 1249.52 |
|  | 128.67 | ± | 3.67 | 216.33 | ± | 19.23 | 216.33 | ± | 19.23 | 216.33 | ± | 19.23 | 128.67 | ± | 3.67 |
| **SNU-387** | 840.67 | ± | 50.78 | 4371.67 | ± | 444.67 | 252.33 | ± | 20.85 | 267.67 | ± | 27.03 | 423.67 | ± | 24.39 |
|  | 154.67 | ± | 8.95 | 184.67 | ± | 17.53 | 184.67 | ± | 17.53 | 184.67 | ± | 17.53 | 154.67 | ± | 8.95 |
| **SNU-423** | 522.67 | ± | 19.10 | 6944.00 | ± | 1116.85 | 485.00 | ± | 25.58 | 572.67 | ± | 11.02 | 5082.33 | ± | 885.23 |
|  | 226.00 | ± | 3.61 | 373.33 | ± | 21.46 | 373.33 | ± | 21.46 | 373.33 | ± | 21.46 | 226.00 | ± | 3.61 |
| **SNU-449** | 257.33 | ± | 1.20 | 264.67 | ± | 4.67 | 202.67 | ± | 10.33 | 246.33 | ± | 12.47 | 4015.33 | ± | 704.73 |
|  | 122.33 | ± | 5.93 | 170.33 | ± | 12.33 | 170.33 | ± | 12.33 | 170.33 | ± | 12.33 | 122.33 | ± | 5.93 |
| **SK-HEP-1** | 590.67 | ± | 49.97 | 581.00 | ± | 22.65 | 300.33 | ± | 10.49 | 409.00 | ± | 17.79 | 4188.00 | ± | 126.81 |
|  | 139.00 | ± | 5.69 | 230.00 | ± | 11.68 | 230.00 | ± | 11.68 | 230.00 | ± | 11.68 | 139.00 | ± | 5.69 |

| **Positive rate** | **CD261 (DR4)** | | | **CD262 (DR5)** | | | **CD263 (DCR1)** | | | **CD264 (DCR2)** | | |
| --- | --- | --- | --- | --- | --- | --- | --- | --- | --- | --- | --- | --- |
| Cell line | Mean ± SEM (%) | | | Mean ± SEM (%) | | | Mean ± SEM (%) | | | Mean ± SEM (%) | | |
| **HepG2** | 18.18 | ± | 4.63 | 99.87 | ± | 0.03 | 7.00 | ± | 0.42 | 8.10 | ± | 1.73 |
| **C3A** | 6.02 | ± | 2.47 | 99.63 | ± | 0.17 | 1.38 | ± | 0.22 | 7.43 | ± | 2.40 |
| **HuH7** | 0.37 | ± | 0.11 | 63.00 | ± | 2.56 | 1.44 | ± | 0.54 | 1.45 | ± | 0.09 |
| **PLC/PRF/5** | 31.10 | ± | 1.63 | 84.23 | ± | 2.70 | 12.88 | ± | 2.68 | 0.32 | ± | 0.11 |
| **SNU-387** | 0.37 | ± | 0.08 | 42.33 | ± | 2.62 | 3.20 | ± | 0.33 | 0.15 | ± | 0.03 |
| **SNU-423** | 0.31 | ± | 0.11 | 11.67 | ± | 1.62 | 2.91 | ± | 0.77 | 0.14 | ± | 0.03 |
| **SNU-449** | 4.25 | ± | 0.95 | 97.63 | ± | 0.46 | 41.30 | ± | 1.97 | 17.57 | ± | 2.93 |
| **SK-HEP-1** | 0.47 | ± | 0.05 | 96.17 | ± | 0.90 | 7.32 | ± | 1.95 | 4.30 | ± | 0.55 |

**Supplementary Table 3b.** Positive rate and MFI for TRAIL receptors in liver cancer cell lines.

| **MFI** | **CD261 (DR4)** | | | **CD262 (DR5)** | | | **CD263 (DcR1)** | | | **CD264 (DcR2)** | | | **Isotype control** | | |
| --- | --- | --- | --- | --- | --- | --- | --- | --- | --- | --- | --- | --- | --- | --- | --- |
| Cell line | Mean ± SEM | | | Mean ± SEM | | | Mean ± SEM | | | Mean ± SEM | | | Mean ± SEM | | |
| **HepG2** | 331.33 | ± | 24.50 | 2401.67 | ± | 201.14 | 295.33 | ± | 7.33 | 267.00 | ± | 15.87 | 135.00 | ± | 3.51 |
| **C3A** | 461.67 | ± | 21.06 | 2804.33 | ± | 173.05 | 373.33 | ± | 26.30 | 411.33 | ± | 28.94 | 180.33 | ± | 23.97 |
| **HuH7** | 427.33 | ± | 11.61 | 1311.67 | ± | 45.12 | 559.67 | ± | 31.75 | 475.33 | ± | 17.65 | 322.33 | ± | 4.63 |
| **PLC/PRF/5** | 590.67 | ± | 29.63 | 1237.67 | ± | 114.90 | 548.00 | ± | 32.72 | 263.67 | ± | 21.33 | 216.33 | ± | 19.23 |
| **SNU-387** | 317.67 | ± | 37.35 | 723.33 | ± | 68.22 | 449.67 | ± | 25.99 | 211.67 | ± | 19.03 | 184.67 | ± | 17.53 |
| **SNU-423** | 588.33 | ± | 52.27 | 902.00 | ± | 35.64 | 892.67 | ± | 33.32 | 413.00 | ± | 18.50 | 373.33 | ± | 21.46 |
| **SNU-449** | 308.33 | ± | 9.21 | 1512.67 | ± | 45.04 | 470.00 | ± | 13.45 | 326.00 | ± | 14.19 | 170.33 | ± | 12.33 |
| **SK-HEP-1** | 378.33 | ± | 12.25 | 2123.33 | ± | 115.84 | 584.67 | ± | 30.43 | 438.33 | ± | 10.48 | 230.00 | ± | 11.68 |

**Supplementary Table 3c.** Positive rate and MFI for CD226 ligands in liver cancer cell lines.

| **Positive rate** | **CD112** | | | **CD155** | | |
| --- | --- | --- | --- | --- | --- | --- |
| Cell line | Mean ± SEM (%) | | | Mean ± SEM (%) | | |
| **HepG2** | 100.00 | ± | 0.00 | 100.00 | ± | 0.00 |
| **C3A** | 99.77 | ± | 0.23 | 99.60 | ± | 0.26 |
| **HuH7** | 99.93 | ± | 0.03 | 99.63 | ± | 0.15 |
| **PLC/PRF/5** | 99.97 | ± | 0.03 | 99.80 | ± | 0.00 |
| **SNU-387** | 99.97 | ± | 0.03 | 99.80 | ± | 0.06 |
| **SNU-423** | 99.87 | ± | 0.03 | 98.07 | ± | 0.38 |
| **SNU-449** | 99.87 | ± | 0.03 | 99.57 | ± | 0.24 |
| **SK-HEP-1** | 99.40 | ± | 0.31 | 98.43 | ± | 0.18 |

| **MFI** | **CD112**  Mean ± SEM | | | **CD155**  Mean ± SEM | | |
| --- | --- | --- | --- | --- | --- | --- |
| Cell line | upper panel: respective molecules | | | | | |
|  | lower panel: isotype control | | | | | |
| **HepG2** | 4421.33 | ± | 351.32 | 3297.33 | ± | 213.72 |
|  | 114.67 | ± | 4.41 | 135.00 | ± | 3.51 |
| **C3A** | 3303.33 | ± | 216.21 | 4184.67 | ± | 461.49 |
|  | 134.00 | ± | 8.96 | 180.33 | ± | 23.97 |
| **HuH7** | 6142.67 | ± | 55.34 | 6004.67 | ± | 320.80 |
|  | 234.00 | ± | 5.57 | 322.33 | ± | 4.63 |
| **PLC/PRF/5** | 6619.67 | ± | 471.79 | 4448.33 | ± | 299.26 |
|  | 128.67 | ± | 3.67 | 216.33 | ± | 19.23 |
| **SNU-387** | 3075.00 | ± | 293.64 | 3776.67 | ± | 243.96 |
|  | 154.67 | ± | 8.95 | 184.67 | ± | 17.53 |
| **SNU-423** | 5467.00 | ± | 108.14 | 5060.33 | ± | 183.62 |
|  | 226.00 | ± | 3.61 | 373.33 | ± | 21.46 |
| **SNU-449** | 5678.33 | ± | 387.06 | 5711.67 | ± | 126.26 |
|  | 122.33 | ± | 5.93 | 170.33 | ± | 12.33 |
| **SK-HEP-1** | 2074.00 | ± | 145.56 | 2511.67 | ± | 119.15 |
|  | 139.00 | ± | 5.69 | 230.00 | ± | 11.68 |

**Supplementary Table 3d.** Positive rate and MFI for GPC3, PD-L1, PD-L2, and CD47 in liver cancer cell lines.

| **Positive rate** | **GPC3** | | | **PD-L1** | | | **PD-L2** | | | **CD47** | | |
| --- | --- | --- | --- | --- | --- | --- | --- | --- | --- | --- | --- | --- |
| Cell line | Mean ± SEM (%) | | | Mean ± SEM (%) | | | Mean ± SEM (%) | | | Mean ± SEM (%) | | |
| **HepG2** | 71.80 | ± | 5.00 | 0.39 | ± | 0.07 | 0.29 | ± | 0.05 | 27.37 | ± | 2.42 |
| **C3A** | 26.50 | ± | 5.40 | 0.32 | ± | 0.08 | 0.18 | ± | 0.03 | 0.20 | ± | 0.04 |
| **HuH7** | 0.52 | ± | 0.24 | 0.34 | ± | 0.11 | 0.14 | ± | 0.04 | 52.47 | ± | 1.78 |
| **PLC/PRF/5** | 12.50 | ± | 1.35 | 0.30 | ± | 0.04 | 0.13 | ± | 0.02 | 79.70 | ± | 3.11 |
| **SNU-387** | 1.63 | ± | 0.14 | 35.63 | ± | 2.28 | 57.93 | ± | 3.78 | 99.67 | ± | 0.15 |
| **SNU-423** | 4.19 | ± | 1.90 | 88.03 | ± | 4.30 | 91.67 | ± | 1.78 | 94.80 | ± | 3.58 |
| **SNU-449** | 7.48 | ± | 1.95 | 51.50 | ± | 3.36 | 16.00 | ± | 1.31 | 99.97 | ± | 0.03 |
| **SK-HEP-1** | 0.51 | ± | 0.20 | 0.16 | ± | 0.01 | 0.75 | ± | 0.10 | 43.37 | ± | 5.59 |

| **MFI** | **GPC3**  Mean ± SEM | | | **PD-L1**  Mean ± SEM | | | **PD-L2**  Mean ± SEM | | | **CD47**  Mean ± SEM | | |
| --- | --- | --- | --- | --- | --- | --- | --- | --- | --- | --- | --- | --- |
| Cell line | upper panel: respective molecules | | | | | | | | | | | |
|  | lower panel: isotype control | | | | | | | | | | | |
| **HepG2** | 576.67 | ± | 114.37 | 155.67 | ± | 6.23 | 164.00 | ± | 6.00 | 359.33 | ± | 9.33 |
|  | 114.67 | ± | 4.41 | 135.00 | ± | 3.51 | 135.00 | ± | 3.51 | 135.00 | ± | 3.51 |
| **C3A** | 333.33 | ± | 36.67 | 200.33 | ± | 23.97 | 211.00 | ± | 27.01 | 243.67 | ± | 23.78 |
|  | 134.00 | ± | 8.96 | 180.33 | ± | 23.97 | 180.33 | ± | 23.97 | 180.33 | ± | 23.97 |
| **HuH7** | 277.00 | ± | 2.08 | 364.00 | ± | 8.72 | 353.33 | ± | 9.84 | 1183.67 | ± | 21.46 |
|  | 234.00 | ± | 5.57 | 322.33 | ± | 4.63 | 322.33 | ± | 4.63 | 322.33 | ± | 4.63 |
| **PLC/PRF/5** | 232.33 | ± | 11.26 | 266.00 | ± | 28.69 | 271.33 | ± | 22.48 | 1289.00 | ± | 104.89 |
|  | 128.67 | ± | 3.67 | 216.33 | ± | 19.23 | 216.33 | ± | 19.23 | 216.33 | ± | 19.23 |
| **SNU-387** | 222.33 | ± | 14.17 | 615.00 | ± | 62.98 | 968.67 | ± | 81.41 | 3512.33 | ± | 364.95 |
|  | 154.67 | ± | 8.95 | 184.67 | ± | 17.53 | 184.67 | ± | 17.53 | 184.67 | ± | 17.53 |
| **SNU-423** | 392.33 | ± | 33.80 | 3569.33 | ± | 636.54 | 3737.67 | ± | 267.11 | 4296.33 | ± | 603.80 |
|  | 226.00 | ± | 3.61 | 373.33 | ± | 21.46 | 373.33 | ± | 21.46 | 373.33 | ± | 21.46 |
| **SNU-449** | 204.00 | ± | 11.72 | 527.67 | ± | 41.95 | 293.67 | ± | 10.49 | 4166.67 | ± | 760.92 |
|  | 122.33 | ± | 5.93 | 170.33 | ± | 12.33 | 170.33 | ± | 12.33 | 170.33 | ± | 12.33 |
| **SK-HEP-1** | 203.33 | ± | 10.65 | 270.67 | ± | 14.84 | 314.67 | ± | 15.93 | 882.33 | ± | 88.94 |
|  | 139.00 | ± | 5.69 | 230.00 | ± | 11.68 | 230.00 | ± | 11.68 | 230.00 | ± | 11.68 |
